# Comparison between static and semi-dynamic models for microcosm biofilm formation on dentin

**DOI:** 10.1590/1678-7757-2018-0163

**Published:** 2019-01-07

**Authors:** Daiana Moreli Soares dos Santos, Juliana Gonçalves Pires, Aline Silva Braga, Priscila Maria Aranda Salomão, Ana Carolina Magalhães

**Affiliations:** 1 Universidade de São Paulo, Faculdade de Odontologia de Bauru, Departamento de Ciências Biológicas, Bauru, São Paulo, Brasil

**Keywords:** Biofilms, Demineralization, Dental caries, Dentin

## Abstract

Microcosm biofilm has been applied to induce carious lesions in dentin. However, no study has been done to compare the impact of the type of model for providing nutrients to microcosm biofilm formation on dentin.

**Objective:**

This study compared the performance of two kinds of models (static and semi-dynamic) on the biofilm formation and the development of dentin carious lesions.

**Material and Methods:**

In both models, biofilm was produced using inoculum from pooled human saliva mixed with McBain saliva for the first 8 h (5% CO_2_ and 37°C). Afterwards, for the static model, the samples were placed in 24-wells microplate containing McBain saliva with 0.2% sucrose, which was replaced at 24 h. In the semi-dynamic model, the samples were submitted to artificial mouth system with continuous flow of McBain saliva with 0.2% sucrose (0.15 ml/min, 37°C) for 10 h a day (for the other 14 h, no flow was applied, similarly to the static model). After 5 days, biofilm viability was measured by fluorescence and dentin demineralization by transverse microradiography.

**Results:**

Biofilm viability was significantly lower for the static compared with semi-dynamic model, while dentin demineralization was significantly higher for the first one (p<0.05). The static model was able to produce a higher number of typical subsurface lesions compared with the semi-dynamic model (p<0.05).

**Conclusions:**

The type of model (static and semi-dynamic) applied in the microcosm biofilm may have influence on it's viability and the severity/profile of dentin carious lesions.

## Introduction

Dental caries is a disease that affects millions of people around the world,^1^ generated by the instability created between the host and the microorganisms from dental biofilm due to the high and frequent consume of sugar, especially sucrose.^2^ Other factors may interfere on biofilm development and increase the risk of root carious lesions, especially for adults and elderly individuals, who present low salivary flow and root exposure due to chronic periodontitis. It has been already established that the prevalence of carious lesions, involving dentin, increases with age.^3^

To better understand the dynamic of biofilm on dentin and to test the protective effect of antimicrobial agents, *in vitro* models of dental caries formation have been applied.^4^ The microcosm biofilm has been considered the biofilm model closest to the *in vivo* reality, making possible to more accurately simulate the complexity of a real dental biofilm *in vitro.* ^5^ ^,^ ^6^ Biofilm models can be further classified according to the availability of nutrients, as: 1) static model, which consists of limited supply of nutrients over time (e.g.: agar plates or multiple well plates); and 2) dynamic model that allows a continuous nutrients supply over time (e.g.: constant depth biofilm fermenter or artificial mouth).^4^ ^–^ ^8^ However, there is no study comparing the impact of the type of model for providing nutrients (static and semi-dynamic) to microcosm biofilm on the development of carious lesion in dentin.

Therefore, this study aimed to compare two models (static and semi-dynamic) regarding the viability of a microcosm biofilm and its capacity of producing carious lesion in dentin. The null hypothesis is that the models do not differ in biofilm viability and capacity of inducing dentin demineralization.

## Material and methods

### Saliva collection

This study was firstly approved by the local Ethical Committee (CAAE: 58330616.7.0000.5417). Saliva was collected from 2 healthy donors only (the amount of saliva was enough for the experiment), who have followed the inclusion criteria: 1) normal salivary flow (stimulated saliva flow >1 ml/min and non-stimulated saliva flow >0.3 ml/min), 2) with previous history of caries, but not active caries (no active white spot and/or cavitated lesions), 3) without gingivitis/periodontitis (gum bleeding or tooth mobility) and 4) who did not ingest antibiotics 3 months before the experiment. The donors were not allowed to brush their teeth in the last 24 h before saliva collection and to ingest food or drinks in the last 2 h before this procedure.^9^ ^,^ ^10^ Saliva was collected under stimulation by chewing a gum for 10 min during the morning. The human saliva pool (70%) was mixed with glycerol (30%) and frozen at −80°C.^9^ ^,^ ^10^

### Tooth sample preparation

Thirty-six dentin samples were prepared from eighteen bovine roots (4 mm x 4 mm, buccal and lingual surfaces) by using a semi-precision cutting machine (Buehler; Lake Bluff, Illinois, USA) and polished using a metallographic polishing machine (Arotec; Cotia, São Paulo, Brazil) and water-cooled silicon-carbide discs (600-grade papers ANSI grit; Buehler; Lake Bluff, Illinois, USA). The average surface roughness was measured using contact profilometer and Mahr Surf XCR 20 software (Mahr; Göttingen, Lower Saxony, Germany), to standard the dentin surface for biofilm formation between the groups. Samples with Ra means <0.2 or >0.4 μm were excluded. The Ra means were further applied for random allocation of the samples into the groups by using the random function of Excel. Two thirds of the root dentin surfaces were protected with wax to obtain control areas for the TMR analysis. The samples were then sterilized using ethylene oxide.

### Microcosm biofilm formation

The microcosm biofilm was formed under two models (18 samples for each model, n=6 *per* biological replicate) for 5 days:

### Static model

For the static model the samples were placed into 24-wells microplate. Human saliva solution was defrosted and mixed with McBain artificial saliva^11^ in a proportion of 1:50.^9^ ^,^ ^10^ During the first 8 h of inoculation, the solution of human saliva and McBain saliva was added to each well containing a root dentin sample (v=1.5 ml), which was incubated at 5% CO_2_ and 37°C. Thereafter, the culture medium was removed and the root dentin samples were washed twice using phosphate-buffered saline (PBS, v=2 ml/well, each time). Fresh culture medium of McBain containing now 0.2% sucrose was added into the wells (v=1.5 ml/well). The microplate were incubated at 5% CO_2_ and 37°C for 16 h, completing the first day of biofilm formation. During the next 4 days, the culture medium was daily removed, the root dentin samples were washed twice using PBS (v=2 ml/well, each time), McBain saliva with 0.2% sucrose was replaced (v=1.5 ml/well), and the microplate were stored at 5% CO_2_ and 37°C.

### Semi-dynamic model

For the semi-dynamic model, the samples were placed into either microplate or artificial mouth. Human saliva solution was defrosted and mixed with McBain artificial saliva^11^ in a proportion of 1:50.^9^ ^,^ ^10^ During the first 8 h of inoculation, the solution of human saliva and McBain saliva was added to each well containing a root dentin sample (v=9 ml). The 6-wells microplate was incubated at 5% CO_2_ and 37°C. Thereafter, the culture medium was removed, and the root dentin samples were washed twice using PBS (v=9 ml/well, each time). Fresh culture medium of McBain saliva containing now 0.2% sucrose was added into the wells (v=9 ml/well). The 6-wells microplate was incubated at 5% CO_2_ and 37°C for 16 h, completing the first day of biofilm formation. During the next 4 days, continuous flow of McBain saliva containing 0.2% sucrose were applied in the artificial mouth for 10 h a day (from 8 am to 6 pm, flow of 0.15 ml/min at 37°C and in an aerobic environment). Overnight (14 h a day), the samples were stored in 6-wells microplate with fresh McBain saliva containing 0.2% sucrose (v=9 ml/well) under 5% CO_2_ and at 37°C. Between the changes, the samples were washed twice using PBS (v=9 ml/well). In this model, 6-wells microplate was applied since the samples should be attached to acrylic disks to be placed into the artificial mouth.

**Figure 1:**
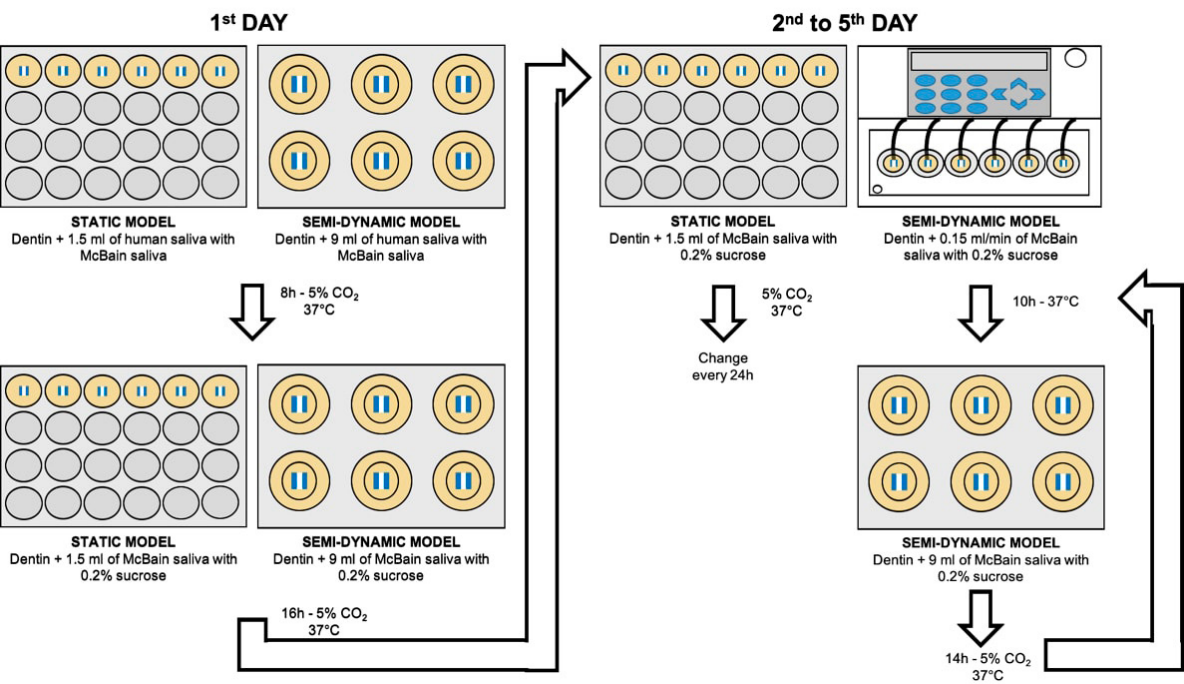
Experimental design

The biofilm cultivation was repeated three-independent times (n=6 independent samples for each type of model *per* replicate). Figure 1 shows the experimental design.

### Bacterial viability analysis

Samples from static and semi-dynamic models (36 in total) were transferred to new 24- and 6- well microplates and exposed to 1 and 9 ml of MTT dye (0.5 mg MTT in 1 ml PBS) *per* well, respectively, for 4 h at 5% CO_2_ and 37°C. The bacteria metabolically active are able to reduce MTT to purple formazan. After 4 h, the dye was removed and 1 and 9 ml of dimethyl sulfoxide (DMSO) was added in each well, respectively, to solubilize the formazan crystals in the absence of light for 30 min. Two hundred microliters from each sample were then transferred to a 96-well microplate, and the absorbance was measured using a microplate reader (Fluostar Optima - BMG Labtech; Ortenberg, Baden-Württemberg, Germany) at 540 nm.^12^ The final values were adjusted to the initial volume.

### Transverse microradiography (TMR)

Dentin samples were sectioned perpendicularly to the wax (to allow the presence of sound and demineralized area in the fragment). Two fragments from each sample (approximately 500 μm thickness each) were manually polished using 600 grit papers, until the approximate thickness of 100-120 μm, and fixed in a sample-holder together with an aluminum calibration step wedge with 14 steps. A microradiograph was taken using an x-ray generator (Softex; Tokyo, Japan) on the glass plate at 20 kV and 20 mA (at a distance of 42 cm) for 13 min. The developed plate was analyzed using a transmitted light microscope fitted with a 20x objective (Zeiss; Oberkochen, Baden-Württemberg, Germany), a CCD camera (Canon; Tokyo, Japan) and a computer. The mineral content was calculated based on the formula described by Angmar, Carlström, Glas^13^ (1963). The integrated mineral loss (ΔZ, %vol.μm) and lesion depth (LD, μm) were calculated as well as the semi-intact surface layer was detected.

### Statistical analysis

Data were statistically analyzed using the software GraphPad InStat for Windows (GraphPad Software; San Diego, California, USA). The normal distribution and homogeneity were checked using Kolmogorov & Smirnov and Bartlett tests, respectively. The biofilm viability data (absorbance) were compared using Mann-Whitney test. ΔZ and LD data were compared using unpaired t test. For the association between the type of model and the percentage of subsurface lesions created, Fisher's Exact Test was done. The level of significance was set at 5% (n=18).

## Results

**Figure 2:**
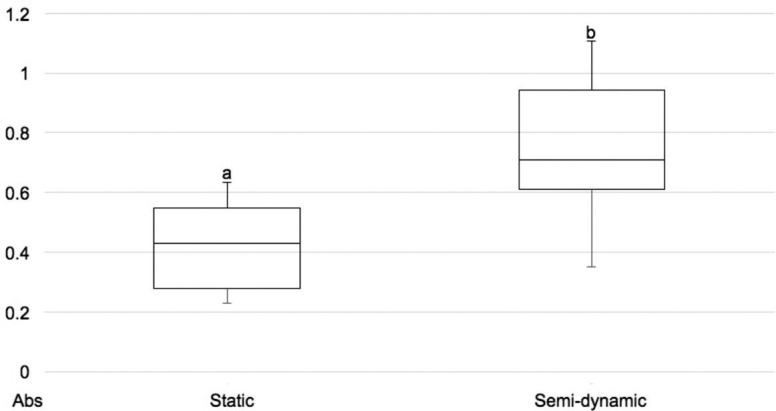
Boxplot of the biofilm viability (absorbance) according to the different models for microcosm biofilm formation. High absorbance values mean high biofilm viability. Different lower script letters indicate statistical significance (Mann-Whitney test, p<0.05)

The biofilm viability was significantly lower for the static model compared with the semi-dynamic model (Figure 2). On the other hand, the static model produced dentin lesions with higher values of the integrated mineral loss and lesion depth compared with the semi-dynamic model (Table 1).

**Table 1:** Mean and standard deviation of the integrated mineral loss (ΔZ, %vol.μm) and the lesion depth (LD, μm) of dentin carious lesions produced using static and semi-dynamic models (microcosm biofilm, 5 days)

Models	ΔZ (%vol.μm)	LD (μm)
Static	4355±685^a^	160.3±16.7^a^
Semi-dynamic	3469±545^b^	129.3±13.2^b^

*Different letters in the same column show significant differences the models (ΔZ: Unpaired T test, p=0.0002. LD: unpaired T test, p<0.0001, n=18). Higher values mean more demineralized lesions

**Figure 3:**
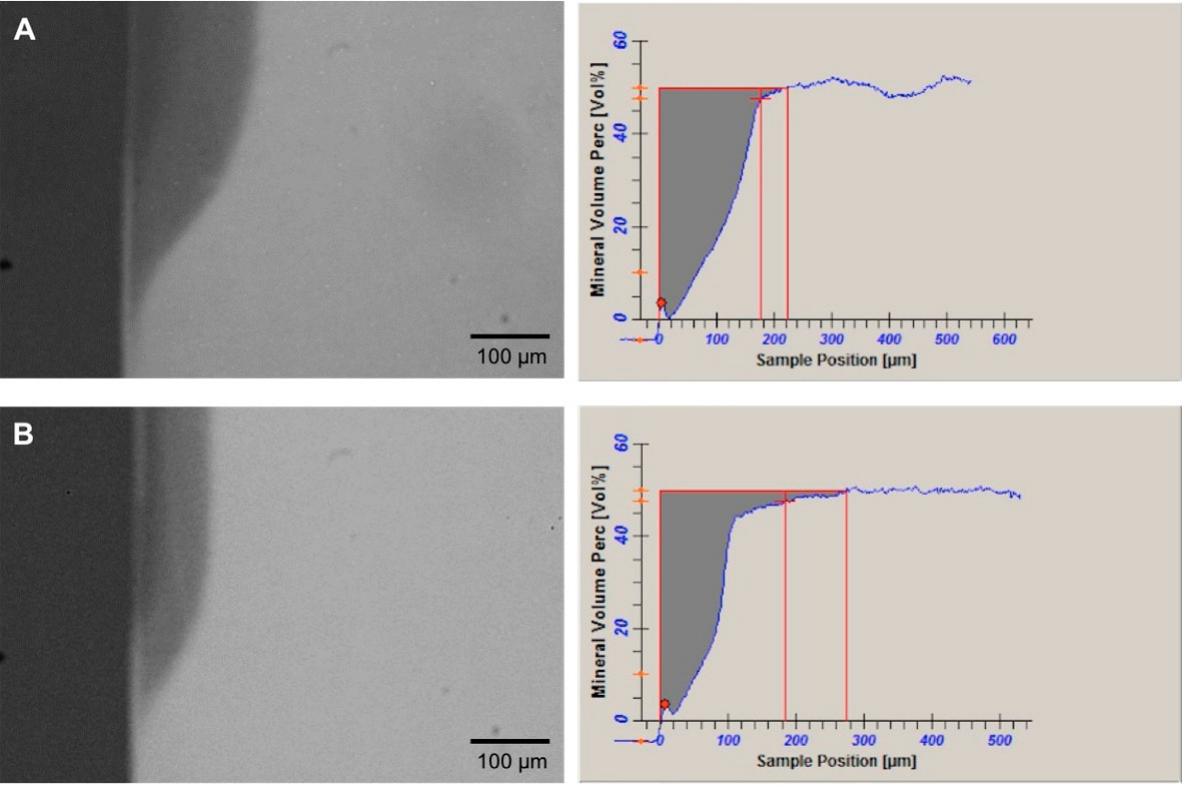
Representative TMR pictures (20x) of the artificial root dentin carious lesions created using microcosm biofilm under A) Static model and B) Semi-dynamic model, showing a more demineralized lesion for the first model

The semi-intact surface layer was often seen in samples from the static model (83%, n=15/18) compared with those from the semi-dynamic model (45%, n=8/18), which means that the static model was able to produce a significant higher number of typical initial subsurface carious lesions (Fisher's Exact Test, p=0.0354). Figure 3 shows representative TMR images of dentin lesions produced by each model.

## Discussion

Despite the advantages of the microcosm biofilm,^4^ ^,^ ^6^ most of the studies have applied this model to produce enamel lesions only,^14^ ^–^ ^16^ highlighting the need of further studies on dentin carious lesion formation. Biofilm formation was induced for 5 days according to Maske, et al.^17^ (2015), after performing a pilot to define the best period to induce initial carious lesions in dentin (without cavitation). Researchers often choose one model to supply nutrients to the microorganisms, being either artificial mouth (an example of dynamic model) or multiple well plates (an example of static model).^4^ ^,^ ^7^ ^,^ ^8^ No attention has been given to compare the impact of the type of model for providing nutrients (static and semi-dynamic) on the viability of microcosm biofilm and the severity of carious lesion formation in dentin.

In this study, biofilm produced using semi-dynamic model showed greater viability compared with those from the static model, which might be due to: 1) continuous flow of nutrients 10 hours *per* day, allowing more microbial growth and/or 2) the capacity of the model to wash metabolites (such as acids) that could be cytotoxic for the bacteria. The second hypothesis may also help to explain the less aggressive dentin lesions induced by this model.

The continuous flow in the semi-dynamic model was simulated only for 10 h, since some periods of flow absence would simulate the nighttime, in which salivary flow rate physiologically decreases to zero. Clarifying that no atmosphere control was provided during 10 h of artificial mouth is important, which might have favored the growth of aerobic and facultative bacteria during this period, justifying the presence of less demineralized lesions. This is a limitation of our artificial mouth, which should be considered in the interpretation of data.

On the other hand, the static model had atmosphere control,^18^ ^,^ ^19^ which may justify the different performances between the models regarding carious lesions formation in dentin. Static model produced deeper dentin lesions than the semi-dynamic model. Also, two hypotheses explain this result: 1) the differences in the type of microorganisms prevalent in both biofilm (probably the number of anaerobic/facultative bacteria was higher for the static model), which should be further investigated and 2) the washing effect provided by the semi-dynamic model, while for the static one the metabolites (acids) could stay in contact with the dentin surface for longer time. Future studies shall be focused on the analysis of biofilm thickness as well as on the differences in microbiome and metabolome between microcosm biofilms formed under both models on dentin.^20^

Other interesting finding of this study was the higher number of typical subsurface lesions produced by the static compared with the semi-dynamic model. This corroborates Owens, et al.^21^ (2017), who showed semi-dynamic biofilm model induced less evident subsurface layer, while Arthur, et al.^22^ (2013) found well-defined subsurface lesions for the static model. The flow in semi-dynamic model may have also washed away free calcium and phosphate from biofilm, reducing their availability to precipitate on the lesion surface.

Considering the limitations of the design and the interpretations of the results, the null hypothesis can be rejected. Both models are able to produce viable cariogenic biofilm and dentin carious lesions; however, semi-dynamic model tends to produce more lesions with loss of surface integrity than the static one, which can be a consequence of the availability of nutrients in each system. The response of both models to antimicrobial agents shall be analyzed in the future, especially concerning the type of microorganisms prevalent in both biofilms and their impact on carious lesions formation in dentin.

## Conclusion

The type of model applied to supply nutrients may have influence on the microcosm biofilm viability and the production of carious lesions in dentin.
